# Kinetics and Product Branching Ratio Study of the CH_3_O_2_ Self-Reaction in the Highly Instrumented Reactor for Atmospheric Chemistry

**DOI:** 10.1021/acs.jpca.2c04968

**Published:** 2022-10-13

**Authors:** Lavinia Onel, Alexander Brennan, Freja F. Østerstro̷m, Ellie Cooke, Lisa Whalley, Paul W. Seakins, Dwayne E. Heard

**Affiliations:** † School of Chemistry, University of Leeds, Leeds, LS2 9JT, United Kingdom; ‡ National Centre for Atmospheric Science, University of Leeds, LS2 9JT, United Kingdom

## Abstract

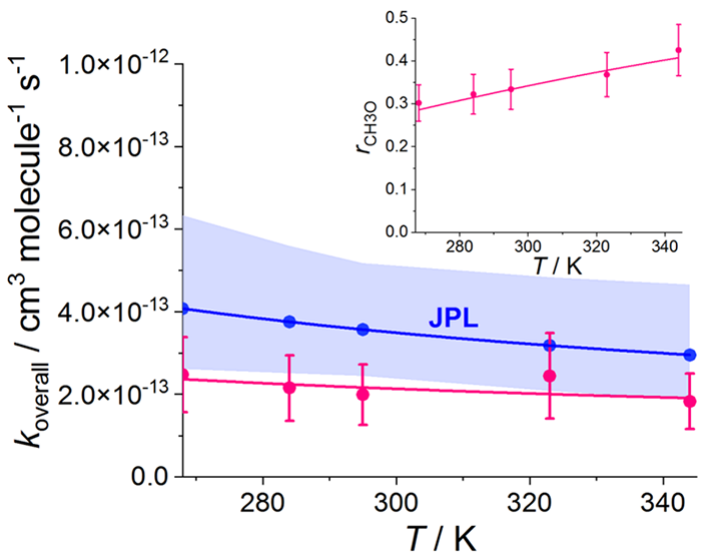

The fluorescence assay by gas expansion (FAGE) method for the measurement of the methyl peroxy radical (CH_3_O_2_) using the conversion of CH_3_O_2_ into methoxy radicals (CH_3_O) by excess NO, followed by the detection of CH_3_O, has been used to study the kinetics of the self-reaction of CH_3_O_2_. Fourier transform infrared (FTIR) spectroscopy has been employed to determine the products methanol and formaldehyde of the self-reaction. The kinetics and product studies were performed in the Highly Instrumented Reactor for Atmospheric Chemistry (HIRAC) in the temperature range 268–344 K at 1000 mbar of air. The product measurements were used to determine the branching ratio of the reaction channel forming methoxy radicals, *r*_CH3O_. A value of 0.34 ± 0.05 (errors at 2σ level) was determined for *r*_CH3O_ at 295 K. The temperature dependence of *r*_CH3O_ can be parametrized as *r*_CH3O_ = 1/{1 + [exp(600 ± 85)/*T*]/(3.9 ± 1.1)}. An overall rate coefficient of the self-reaction of (2.0 ± 0.9) × 10^–13^ cm^3^ molecule^–1^ s^–1^ at 295 K was obtained by the kinetic analysis of the observed second-order decays of CH_3_O_2_. The temperature dependence of the overall rate coefficient can be characterized by *k*_overall_ = (9.1 ± 5.3) × 10^–14^ × exp((252 ± 174)/*T*) cm^3^ molecule^–1^ s^–1^. The found values of *k*_overall_ in the range 268–344 K are ∼40% lower than the values calculated using the recommendations of the Jet Propulsion Laboratory and IUPAC, which are based on the previous studies, all of them utilizing time-resolved UV–absorption spectroscopy to monitor CH_3_O_2_. A modeling study using a complex chemical mechanism to describe the reaction system showed that unaccounted secondary chemistry involving Cl species increased the values of *k*_overall_ in the previous studies using flash photolysis to initiate the chemistry. The overestimation of the *k*_overall_ values by the kinetic studies using molecular modulation to generate CH_3_O_2_ can be rationalized by a combination of underestimated optical absorbance of CH_3_O_2_ and unaccounted CH_3_O_2_ losses to the walls of the reaction cells employed.

## Introduction

Methyl peroxy (CH_3_O_2_) radicals are key species in atmospheric oxidation^1^ and the combustion of volatile organic compounds.^2,3^ The chemistry of CH_3_O_2_ in the troposphere is typically dominated by the reaction with NO, particularly in environments influenced by anthropogenic NO_*x*_ emissions (reaction R1). The reaction is a critical step in the tropospheric production of ozone in the presence of NO and converts NO into NO_2_:
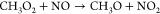
R1The subsequent reaction of CH_3_O with O_2_ produces HO_2_, which then oxidizes another NO to NO_2_:
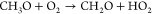
R2
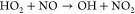
R3During the daytime the NO_2_ photolysis is the dominant tropospheric source of O_3_. In addition, the CH_3_O_2_ + NO reaction leads to the propagation of HO_*x*_ and NO_*x*_ radical chains. However, under low NO_*x*_ levels the self-reaction of CH_3_O_2_ and the reactions of CH_3_O_2_ with HO_2_ and other organic peroxy (RO_2_) species are significant losses of CH_3_O_2_ and terminate the radical chain. The CH_3_O_2_ self-reaction occurs through two channels, reactions R4.a and R4.b:^4^
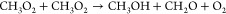
R4.a
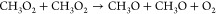
R4.bThe methoxy radicals generated by channel R4.b subsequently react with oxygen (reaction R2) to form CH_2_O and HO_2_.

Despite its importance, the reported values for the rate coefficient of reaction R4, *k*_4_, at room temperature lie in a wide range from (2.7–5.2) × 10^–13^ cm^3^ molecule^–1^ s^–1^,^5^ with IUPAC^5^ and the Jet Propulsion Laboratory (JPL)^6^ giving 20–40% and 40–50%, respectively, uncertainties at the 2σ level for *k*_4_ in the temperature range 270–350 K. The previous kinetic studies used either the flash photolysis (FP) technique^7−11^ or the molecular modulation (MM) method^12−14^ to generate CH_3_O_2_ radicals, which were coupled to time-resolved UV–absorption spectroscopy to detect CH_3_O_2_ at fixed wavelengths in the range ∼210–270 nm (typically at 250 nm). As UV-absorption is a relatively insensitive technique, the detection limits of CH_3_O_2_ were high, for example around 4 × 10^12^ molecules cm^–3^,^8,11^ and the UV-absorption studies used high initial concentrations of CH_3_O_2_, 10^13^–10^14^ molecules cm^–3^ orders of magnitude.^7−11^

The kinetic studies of the CH_3_O_2_ self-reaction used photolytic mixtures of CH_4_/Cl_2_/O_2_ to generate CH_3_O_2_.^7−14^ CH_3_O formed by the reaction R4.b is rapidly removed via the reaction with O_2_ (reaction R2) in high concentrations (10^17^–10^18^ molecules cm^–3^ orders of magnitude)^7−14^ to generate HO_2_, which quickly reacts further with another CH_3_O_2_ radical (reaction R5).
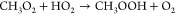
R5As each HO_2_ radical consumes rapidly one CH_3_O_2_ species on the time scale of the CH_3_O_2_ self-reaction, the determination of the overall rate coefficient of the reaction R4, *k*_4_, requires knowledge of the branching ratios for reaction R4 (eq 1)^7,11^
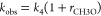
1where *k*_obs_ is the second-order observed rate coefficient and *r*_CH3O_ is the branching ratio of the reaction channel producing CH_3_O (reaction R4.b).

The branching ratios in the CH_3_O_2_ self-reaction were the subject of a number of experimental studies performed from the mid 1970s to 1990 inclusive, which were followed by the study of Tyndall et al. in 1998.^15^ The studies used photolysis of CH_4_/Cl_2_/O_2_ or (CH_3_)_2_N_2_/O_2_ and either end product detection employing mass spectrometry (MS),^16^ GC-MS,^17^ and infrared spectroscopy,^15,18−20^ or kinetic measurements using time-resolved UV–absorption spectroscopy.^7^

Some of the early studies reported a third channel of the self-reaction leading to CH_3_OOCH_3_ (reaction R4.c) with an insignificant contribution to the overall reaction rate coefficient at all temperatures—such as ≤0.08 at 297 K^19^ and ≤0.07 at 298 K^18^—with other studies finding no evidence for any contribution of peroxide formation.^15,20^
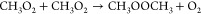
R4.cIUPAC^5^ and JPL^6^ use the evaluation of Tyndall et al.^21^ that recommends considering reactions R4.a and R4.b as the sole reaction channels of the self-reaction.

The majority of the branching ratios studies were carried out at room temperature^15,16,18−20^ and resulted in a range of values for the branching ratio of the reaction channel leading to CH_3_O (reaction R4.b): *r*_CH3O_ = 0.22–0.45. Tyndall et al.^21^ revised the room temperature results to obtain *r*_CH3O_ = 0.37 ± 0.06 at 298 K, which is recommended by IUPAC^5^ and JPL^6^. There have been two experimental studies of the temperature dependence of the branching ratios, which were conducted over different temperature ranges.^7,20^ Lightfoot et al.^7^ found a positive temperature dependence for *r*_CH3O_ between 388 and 573 K using flash photolysis in combination with time-resolved UV–absorption spectroscopy. Horie et al.^20^ performed the only branching ratio experimental study covering temperatures below room temperature using matrix isolation Fourier transform infrared spectroscopy. The results were obtained from 223–333 K to show a positive temperature dependence for *r*_CH3O_. Tyndall et al.^21^ combined the results of Lightfoot et al.^7^ and Horie et al.^20^ with their recommended value at 298 K, *r*_CH3O_ = 0.37 ± 0.06, and results published prior to 1990 to describe the ratio of the rate coefficients of the two reaction channels R4.a and R4.b as. The evaluation of Tyndall et al.^21^ is recommended by JPL.^6^

This work reports on the determination of the branching ratios and the overall rate coefficient of the CH_3_O_2_ self-reaction in the temperature range of 268–344 K at 1000 mbar of synthetic air. The kinetic and branching ratio measurements were performed in the Highly Instrumented Reactor for Atmospheric Chemistry (HIRAC). Fourier Transform Infrared (FTIR) spectroscopy was employed to monitor the time profiles of the concentrations of CH_2_O and CH_3_OH produced by the CH_3_O_2_ self-reaction to determine the branching ratios of the two reaction channels R4.a and R4.b.

The fluorescence assay by gas expansion (FAGE) method for the selective and sensitive detection of CH_3_O_2_ radicals was used to study the kinetics of the self-reaction.^22,23^ The method involves the titration of CH_3_O_2_ to CH_3_O by reaction with added NO, followed by the detection of the resultant CH_3_O by off-resonant LIF with laser excitation at *ca*. 298 nm.^22^ The FAGE instrument was calibrated for CH_3_O_2_ using the 184.9 nm photolysis of water vapor in air to generate OH followed by the conversion of OH to known concentrations of CH_3_O_2_ by reaction with CH_4_ and O_2_.^22,23^ The 184.9 nm photolysis of water vapor is a well-established method of FAGE calibration for OH and HO_2_.^24−26^ The FAGE method for the CH_3_O_2_ detection has been validated previously using the direct and absolute near-IR Cavity Ring Down Spectroscopy (CRDS) method to detect CH_3_O_2_.^23^

FAGE measurements were carried out in the HIRAC chamber to determine the observed rate coefficient of the CH_3_O_2_ self-reaction, *k*_obs_, at 1000 mbar and temperatures in the range of 268–344 K. Using *k*_obs_ and the branching ratio of the reaction channel leading to methoxy radicals, *r*_CH3O_, the overall rate coefficient of the self-reaction, *k*_4_, was derived. This is the first kinetic study of the CH_3_O_2_ self-reaction using a different detection method to that of UV–absorption spectroscopy.

## Experimental Section

### CH_3_O_2_ Generation in HIRAC

The HIRAC chamber is a stainless steel cylinder with an internal volume of ∼2.25 m^3^ and has been described in detail elsewhere.^22,23,27,28^ Four circulation fans mounted in pairs at each end of HIRAC are used to homogenize the gas mixture contained in the chamber. The photochemistry is initiated by eight UV lamps, each of them housed in a quartz tube. The quartz tubes are mounted radially inside the chamber (aligned parallel to the chamber longitudinal axis). In order to perform experiments at temperatures different to the room temperature a thermofluid (HUBE6479 DW-therm oil) is circulated from a high capacity thermoregulator (Huber Unistat 390W) through a series of stainless steel channels welded to the outside of the chamber. To ensure that the temperature is homogeneous within the chamber, the layout of these channels is evenly distributed on the chamber outer surface and HIRAC is lagged in a 20-mm-thick expanded neoprene.

The experiments were carried out at 268, 284, 295, 323, and 344 K and 1000 mbar of synthetic air obtained by mixing high purity oxygen (BOC, > 99.999%) and nitrogen (BOC, > 99.998%) in the ratio of O_2_:N_2_ = 1:4. CH_4_ (BOC, CP grade, 99.5%) and Cl_2_ (Sigma-Aldrich, ≥ 99.5%) were delivered to the chamber. Initial reagent concentrations in HIRAC were [CH_4_] = (2.0–3.0) × 10^17^ molecules cm^–3^ and [Cl_2_] = (0.3–5.5) × 10^14^ molecules cm^–3^. After adding the reagents into the chamber, the lamps (Phillips, TL-D36W/BLB, λ = 350–400 nm) were turned on to generate CH_3_O_2_ by Cl_2_ photolysis at ∼365 nm (reaction R6) followed by reactions R7 and R8. In the kinetic experiments the lamps were turned on for about 5 min, and then they were turned off to record the generated CH_3_O_2_ kinetic decay. In the experiments performed to determine the product branching ratio the lamps were turned on to measure CH_3_OH and CH_2_O using FTIR spectroscopy for a typical time of 20 min.
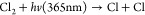
R6
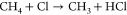
R7
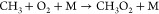
R8

### Fourier Transform Infrared (FTIR) Measurements

An *in situ* multipass FTIR (Bruker IFS66) arrangement along the long axis of HIRAC was used to measure the concentrations of CH_2_O and CH_3_OH produced during the times with the lamps turned on. The multipass Chernin arrangement within the chamber was optimized for 72 internal reflections giving an approximate total path length of 128.5 m.^27,29^ IR spectra were recorded every 30–60 s as the average of 30–100 scans at 1 cm^–1^ resolution. The concentration–time profiles for CH_2_O and CH_3_OH were obtained using the absorption at around 1740 cm^–1^ due to the stretch of the C=O bond of CH_2_O and at around 1030 cm^–1^ due to the C–O stretch of CH_3_OH and using reference spectra taken of formaldehyde and methanol. Reference spectra were taken delivering CH_2_O and CH_3_OH in known concentrations to the chamber under the same conditions as those used for the CH_3_O_2_ self-reaction experiments. The reference compound, either CH_2_O or CH_3_OH, was delivered in the vapor phase by direct heating of either liquid CH_3_OH or *para*-formaldehyde powder in a glass finger connected to a one liter stainless steel cylinder to achieve a gas pressure of a few mbar. Then the gas was delivered from the cylinder to the chamber using a flow of N_2_ (*p*_N2_ = 2000 mbar).

The reference spectra of CH_2_O and CH_3_OH were fitted to the observed IR absorbance recorded as a function of wavelength (λ) and time (*t*), *A*_obs_λ,*t*__, between ∼1600–1900 cm^–1^ and ∼900–1120 cm^–1^, respectively, at each time point to determine the changes to concentrations of CH_2_O and CH_3_OH vs time during the period of time with the lamps switched on (eq 2).
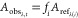
2Here *A*_ref_λ(*i*)__ is the reference IR absorbance of species *i* as a function of λ and , where [*i*]_t_ is the concentration of species *i* at reaction time *t* and [*i*]_ref_ is the concentration of species *i* giving the reference spectrum. The species *i* is CH_2_O in the fit using λ ≅ 1600–1900 cm^–1^ and CH_3_OH in the fit over ∼900–1120 cm^–1^. The concentrations [*i*]_t_ (*i* = CH_2_O or CH_3_OH) were then derived (eq 3).
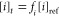
3

### FAGE Instrument and Calibration for CH_3_O_2_

Details on the HIRAC FAGE instrument are provided in previous publications.^22,23,30^ The instrument sampled gas through a 1-mm-diameter pinhole mounted on one end of a 50-mm-i.d. flow tube at a rate of ∼3 slpm. The pressure inside the sampling tube was maintained at 3.3 mbar for a chamber pressure of 1000 mbar of synthetic air. A CH_3_O fluorescence detection cell was integrated in the tube at ∼600 mm distance from the pinhole. About 25 mm prior to the detection cell, high purity NO (BOC, N2.5 nitric oxide) was injected at 2.5 sccm using a mass flow controller (Brooks 5850S) into the center of the gas flow to convert CH_3_O_2_ radicals into CH_3_O. CH_3_O radicals were subsequently detected by LIF spectroscopy, directing laser light at λ_online_ ≅ 297.79 nm to excite the A^2^A_1_(ν_3_′ = 3) ← X^2^E(ν_3_″ = 0) transition of CH_3_O with a 5 kHz pulse repetition frequency through the cell at a right angle to the gas flow. The off-resonant red-shifted LIF (320–430 nm) was monitored using photon counting. The laser background was measured at a wavelength of λ_online_ + 2.5 nm and then subtracted to obtain the fluorescence signal.

The FAGE technique requires calibration to convert the measured fluorescence signal into CH_3_O_2_ concentration. The calibration procedure has been described in detail previously^22,23^ and hence only the important points are presented here. OH radicals were generated photolyzing water vapor in synthetic air at 184.9 nm to react with methane in excess (BOC, CP grade, 99.5%) to generate CH_3_O_2_. The produced air/radical mixture was then sampled by the FAGE instrument. The concentration of CH_3_O_2_ was determined using eq 4.
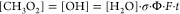
4Here σ is the absorption cross section of water vapor at 184.9 nm, (7.2 ± 0.2) × 10^–20^ cm^2^ molecule^–1^;^31,32^ Φ is the photodissociation quantum yield of OH at 184.9 nm (unity), *t* is the photolysis time, and *F* is the lamp flux at 184.9 nm, which was varied to generate a range of CH_3_O_2_ radical concentrations. The product *F* × *t* was determined employing chemical actinometry.^28^

The FAGE calibration factor was utilized to determine [CH_3_O_2_] in the HIRAC experiments:
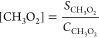
5where *S*_CH_3_O_2__ (counts s^–1^ mW^–1^) is the recorded signal. Previous studies have shown that the FAGE sensitivity toward OH does not depend on the chamber temperature in the range 263–344 K,^33^ and thus the calibration factor determined at a room temperature of 295 K, *C*_CH_3_O_2__ = (5.0 ± 1.7) × 10^–10^ counts cm^3^ molecule^–1^ s^–1^ mW^–1^, was used in the kinetic analysis at all temperatures.

## Results

### Product Branching Ratios in the CH_3_O_2_ Self-Reaction

To determine the branching ratios in the CH_3_O_2_ self-reaction (reactions R4.a and R4.b) the time profiles of the concentrations of the self-reaction products CH_3_OH and CH_2_O generated by turning the lamps on were recorded employing FTIR spectroscopy at a time intervals of 30–60 s. Over the first few minutes of the reaction [CH_3_OH] and [CH_2_O] increased linearly in time, showing that the removal of CH_3_OH and CH_2_O by secondary reactions is negligible. Figure S5 (Supporting Information) shows that at later reaction times [CH_3_OH] vs time and [CH_2_O] vs time curve down due to the secondary reactions of the products, predominantly the CH_3_OH + Cl and CH_2_O + Cl reactions. Numerical simulations carried out using a chemical mechanism described in the Supporting Information for the reaction system at 298 K show that, following about 25 s induction time, [CH_3_OH] and [CH_2_O] increase linearly during the first few minutes of the reaction (Figure S6).

Using the integrating rate ratio for the two parallel reactions R4.a and R4.b, eq 6 is obtained.
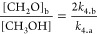
6Here [CH_2_O]_b_ is the concentration of CH_2_O formed by reaction R4.b followed by reaction R2. Taking into account that FTIR measures the sum of the concentrations of CH_2_O produced by the two channels, i.e., [CH_2_O]_a_ produced by reaction R4.a and [CH_2_O]_b_ obtained by reactions R4.b + R2, and [CH_2_O]_a_ = [CH_3_OH], eq 7 is derived from eq 6. Equation 7 was employed in previous FTIR product studies of the CH_3_O_2_ self–reaction.^15,19^
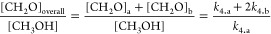
7Using eq 7 the branching ratio of the reaction channel R4.b, which produces CH_3_O, is given by
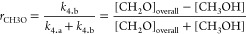
8

The branching ratio *r*_CH3O_ was determined using [CH_2_O]_overall_ and [CH_3_OH] measured at early reaction times, when the product concentrations increased linearly in time (Figure S5 in the Supporting Information). A number (6–20) of values of *r*_CH3O_ were obtained at each temperature, 268, 284, 295, 323, and 344 K. Figure S7 (Supporting Information) shows that there was no trend with time in the extracted values over the initial few minutes used to determine *r*_CH3O_, and thus the secondary reactions of CH_2_O and CH_3_OH can be neglected in the analysis.

**Figure 1:**
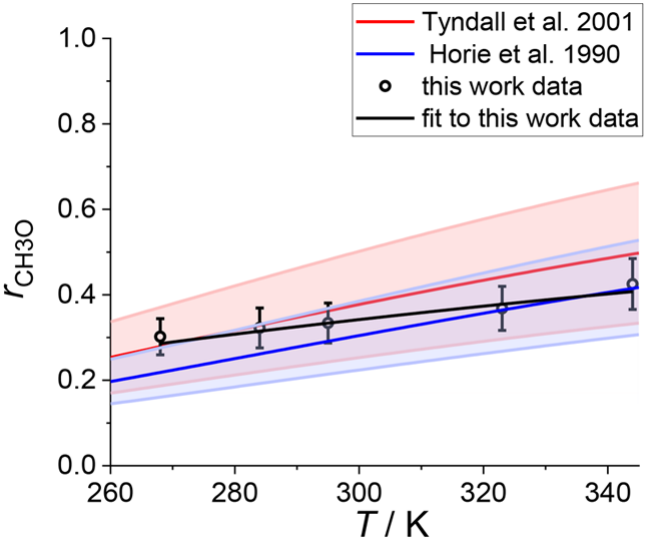
Product branching ratio for the channel giving CH_3_O of the CH_3_O_2_ self-reaction, *r*_CH3O_, as a function of temperature, *T*. The data obtained in this work are shown as open circles with the fit result, 1/{1 + [exp(600 ± 85)/*T*]/(3.9 ± 1.1)}, shown in black. The blue line and shading show the result of Horie et al.,^20^*r*_CH3O_ = 1/{1 + [exp(1131 ± 30/*T*)]/(19 ± 5)} and the red line and shading show *r*_CH3O_ vs *T* derived from *k*_4.b_/*k*_4.a_ vs *T* evaluated by Tyndall et al.,^21^ (26.2 ± 6.6) × exp[−(1130 ± 240)/*T*] and recommended by the Jet Propulsion Laboratory evaluation.^6^ All the errors are given at the 2σ level.

The mean values of *r*_CH3O_ are shown in Figure 1 and Table S2 (Supporting Information). The results show a positive temperature dependence which can be characterized by *r*_CH3O_ = 1/{1 + [exp(600 ± 85)/*T*]/(3.9 ± 1.1)}, i.e., *k*_4.b_/*k*_4.a_ = (3.9 ± 1.1) × exp(−600 ± 85)/*T*. There have been two temperature dependence studies of the branching ratios previously, in the range 388–573 K^7^ and between 223–333 K.^20^ The result obtained by Horie et al.,^20^*r*_CH3O_ = 1/{1 + [exp(1131 ± 30/*T*)]/(19 ± 5)}, is shown in Figure 1, as the temperature range used by these authors overlaps with the range of temperatures where the measurements reported in the present work were carried out. In addition, Figure 1 shows *r*_CH3O_ derived from the evaluation of Tyndall et al.,^21^*k*_4.b_/*k*_4.a_ = (26.2 ± 6.6) × exp[−(1130 ± 240)/*T*], which is recommended by the Jet Propulsion Laboratory (JPL) evaluation.^6^

The values found by this work have overlapping error limits with the results reported by Horie et al.^20^ and the values given by the recommendation of Tyndall et al.^21^ at the 2σ level. However, the temperature dependence measured by Horie et al.^20^ and the temperature dependence recommended by Tyndall et al.^21^ are steeper than the increase in the value of *r*_CH3O_ with the temperature found in this study. The result at 295 K, *r*_CH3O(this work)_ = 0.34 ± 0.05, is between the result of Horie et al.,^20^*r*_CH3O(Horie et al.)_ = 0.29 ± 0.08, and the value recommended by Tyndall et al.,^21^*r*_CH3O(Tyndall at al.)_ = 0.36 ± 0.12 (uncertainties at 2σ level).

The evaluation of Tyndall et al.^21^ is based on the results of Horie et al.^20^ between 223–333 K, Lightfoot et al.^7^ in the range 388–573 K, results published prior to 1990 at *T* ≥ 373 K, and the result of the evaluation of the room temperature values, *r*_CH3O_(298 K) = 0.37 ± 0.06.^15^ The study of Horie et al.^20^ is the single experimental study performed at temperatures in a range around room temperature, i.e., (293_–70_^+40^) K. The results of the present study agree well with the values reported by Horie et al.^20^ at 323 and 344 K (Figure 1). However, going down in temperature *r*_CH3O(this work)_ is increasingly higher than *r*_CH3O(Horie et al.)_.^20^

Horie et al.^20^ carried out flow tube experiments using photolysis of CH_4_/Cl_2_/O_2_ mixtures to measure the ratio [CH_2_O]/[CH_3_OH], where CH_2_O and CH_3_OH were produced by the CH_3_O_2_ self-reaction, employing matrix isolation Fourier transform infrared spectroscopy. The authors performed numerical simulations based on a complex model to vary the rate coefficients of the self-reaction channels to match the measured values for [CH_2_O]/[CH_3_OH]. The authors found no evidence for the formation of CH_3_OOCH_3_ by the reaction channel R4.c. However, two sets of numerical simulations were performed: assuming a branching ratio of 0.1 for reaction R4.c and excluding reaction R4.c from the chemical mechanism used in the numerical simulations. Figure 1 shows the reported temperature dependence derived averaging the results generated by the two sets of simulations.^20^ Considering a zero contribution for reaction R4.c, in line with the present recommendations,^5,6^ the reported values of *r*_CH3O(Horie et al.)_ increase by 5% over the temperature range 268–344 K and are lower than *r*_CH3O(this work)_ by 7% at 295 K, 14% at 284 K, and 22% at 268 K.

The present work measured [CH_2_O] and [CH_3_OH] *in situ* to determine *r*_CH3O_ (eq 8), while Horie et al.^20^ trapped the reaction products outside the reaction cell in a CO_2_ matrix at 50 K to analyze them by IR spectroscopy to obtain [CH_2_O]/[CH_3_OH], which was then used in the determination of *r*_CH3O_. The concentrations of CH_2_O produced by the self-reaction were corrected taking into account CH_2_O formed in the matrix using a correction factor less than 10%. The lower values obtained for *r*_CH3O(Horie et al.)_ relative to *r*_CH3O(this work)_ at *T* ≤ 295 K can be explained by a process leading to the CH_2_O removal enhanced by reducing the reaction temperature which was not included in the reaction mechanism employed in the analysis performed by the authors.^20^ Horie et al.^20^ reported evidence of aerosol formation at 213 K resulting in unaccounted removal of CH_2_O leading to a value of [CH_2_O]/[CH_3_OH] lower than unity, a result which was not expected based on the reaction mechanism; the results obtained at 213 K were thus excluded from the analysis. The experiments below room temperature used in the determination of *r*_CH3O_—i.e., in the range 223–298 K—were reported “free” of aerosols. However, [CH_2_O] and [CH_3_OH] in the experiments were relatively large, a few times higher than in the present work, increasing the potential of oligomers/particle formation at low temperatures.

### Kinetics of the CH_3_O_2_ Self-Reaction

**Figure 2:**
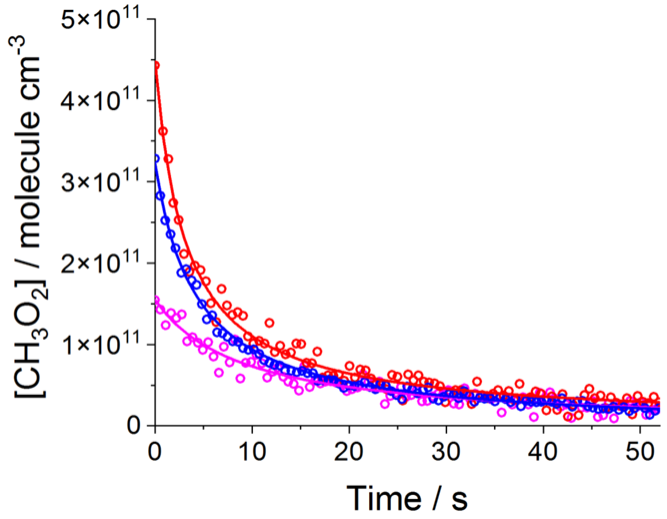
Examples of observations of CH_3_O_2_ (open circles) and fits to the data (solid lines) generated employing eq 9. The experiments used Cl_2_/CH_4_/O_2_ and black lamps (see main text for details); 323 K and 1000 mbar mixture of N_2_:O_2_ = 4:1. At time zero the lamps were turned off. [CH_4_]_0_ = 2.5 × 10^17^ molecules cm^–3^ for all the kinetic decays. Initial Cl_2_ concentrations: 3.3 × 10^14^ molecules cm^–3^ (red), 2.4 × 10^14^ molecules cm^–3^ (blue), and 7.5 × 10^13^ molecules cm^–3^ (magenta).

Figure 2 shows examples of CH_3_O_2_ decay generated by turning the HIRAC lamps off following the production of CH_3_O_2_ by the Cl atom initiated oxidation of CH_4_ in the presence of O_2_ (reactions R6 and R7) at 323 K. Kinetic decays were obtained in a similar fashion at all temperatures. The CH_3_O_2_ decays at each temperature, 268, 284, 295, 323, and 344 K, measured using FAGE were fitted simultaneously to the integrated second-order rate law equation describing the CH_3_O_2_ self-reaction (R4)):
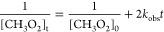
9where [CH_3_O_2_]_*t*_ is the methyl peroxy concentration at reaction time *t*, [CH_3_O_2_]_0_ is the initial concentration when the lights are switched off and *k*_obs_ is the observed rate coefficient. In line with previous analysis,^23^ this work found that the loss of CH_3_O_2_ to the walls of HIRAC was negligible over the time scale of 0.5–1 min of the kinetic measurements at all temperatures employed, and hence a wall loss was not included in the kinetic analysis. Typically about 20 CH_3_O_2_ decays were fitted simultaneously at each temperature to obtain *k*_obs_ with the results shown in Table 1.

**Table 1:** Observed Rate Coefficient, *k*_obs_ for the CH_3_O_2_ Self-Reaction Measured in This Work

Temperature/K	*k*_obs_ (this work)^a^/cm^3^molecule^–1^ s^–1^
268	(3.2 ± 1.1) × 10^–13^
284	(2.9 ± 1.0) × 10^–13^
295	(2.7 ± 0.9) × 10^–13^
323	(3.4 ± 1.4) × 10^–13^
344	(2.6 ± 0.9) × 10^–13^

a errors are 2σ.

The observed rate coefficient is larger than the second-order rate coefficient of just the CH_3_O_2_ recombination reaction (R4), *k*_4_, as the methoxy radicals generated by channel R4.b react rapidly with molecular oxygen, which is present in large excess, 5 × 10^18^ molecules cm^–3^, to produce HO_2_ (reaction R2), which in turn reacts with another CH_3_O_2_ radical (reaction R5). As each HO_2_ radical consumes one CH_3_O_2_ species (reaction R5) on the time scale of reaction R4, *k*_4_ is derived from *k*_obs_ as follows:^7,11^
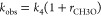
1where *r*_CH3O_ is the branching ratio for the reaction channel R4.b. The applicability of eq 1 in the analysis of the kinetic data generated by the HIRAC experiments was demonstrated by modeling the observed temporal decays using a variety of CH_3_O_2_ and HO_2_ concentrations representative for the HIRAC experiments and incorporating a heterogeneous loss of HO_2_ in the model relevant for the experiments:^22,23^*k*_loss_ = 0.01–0.1 s^–1^. The results showed that the removal of HO_2_ by wall loss is negligible and thus can be excluded from the model.^22^

**Figure 3:**
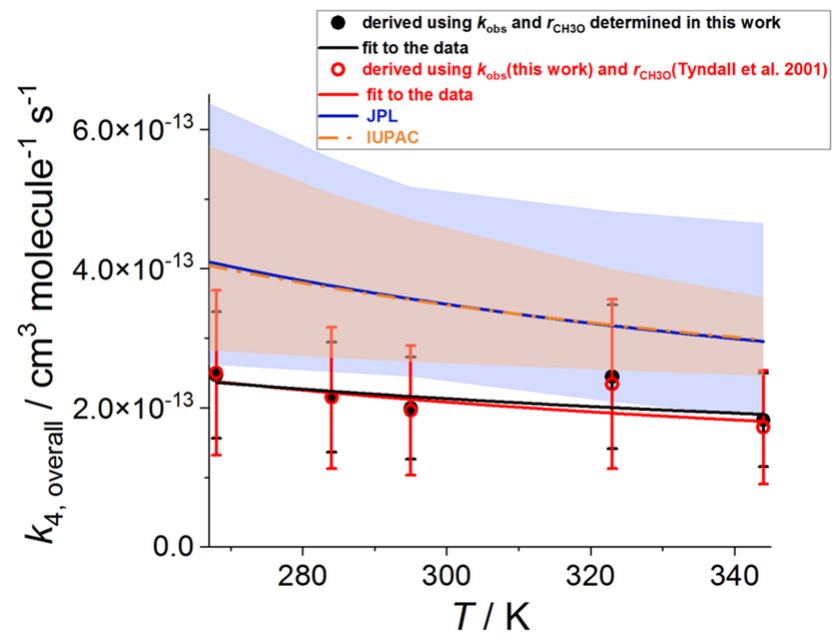
Temperature dependence of the overall rate coefficient of the CH_3_O_2_ self-reaction (R4), *k*_4_. The data generated using *k*_obs_ and *r*_CH3O_ obtained in this work (black circles) are plotted with the data generated using *k*_obs_ measured by this work and *r*_CH3O_ given by the evaluation of Tyndall et al.^21^ (open red circles) and *k*_4_ recommended by JPL (blue line and shading)^6^ and IUPAC (orange dashed line and shading).^5^ The blue and orange shadings show the 2σ uncertainties in the JPL and IUPAC recommendations. The results of the fit to the data are *k*_4_ = (9.1 ± 5.3) × 10^–14^ × exp((252 ± 174)/*T*) cm^3^ molecule^–1^ s^–1^ (black line) and *k*_4_ = (6.8 ± 4.1) × 10^–14^ × exp((335 ± 179)/*T*) cm^3^ molecule^–1^ s^–1^ (red line). The parametrization of the temperature dependence of *k*_4_ recommended by JPL and IUPAC are *k*_4_(JPL) = 9.5 × 10^–14^ × exp(390/*T*) cm^3^ molecule^–1^ s^–1^^6^ and *k*_4_(IUPAC) = 1.03 × 10^–13^ × exp((365)/*T*) cm^3^ molecule^–1^ s^–1^.^5^

Figure 3 shows the determined temperature dependence for *k*_4_. At all temperatures employed, the values of *k*_4_ obtained using both *k*_obs_ and *r*_CH3O_ determined in this work are practically the same as the *k*_4_ values obtained using *k*_obs_ determined in this work and *r*_CH3O_ given by the evaluation of Tyndall et al.,^21^ which is recommended by JPL.^6^ Using the value of *r*_CH3O_ = 0.34 ± 0.05 determined at 295 K in this work the rate coefficient of the overall reaction *k*_4_(295 K) = (2.0 ± 0.7) × 10^–13^ cm^3^ molecule^–1^ s^–1^ (uncertainties at 2σ level). Using the value of *r*_CH3O_(295 K) = 0.36 ± 0.12 recommended by Tyndall et al.^21^ does not change the result at this level of precision: *k*_4_(295 K) = (2.0 ± 0.9) × 10^–13^ cm^3^ molecule^–1^ s^–1^ (uncertainties quoted at 2σ level). The negative temperature dependence obtained employing *r*_CH3O_ determined in this work can be characterized by *k*_4_ = (9.1 ± 5.3) × 10^–14^ × exp((252 ± 174)/*T*) cm^3^ molecule^–1^ s^–1^. Figure 3 compares the result of this work with *k*_4_ vs *T* recommended by JPL and IUPAC. The JPL and IUPAC recommendations are similar to each other: *k*_4_(JPL) = 9.5 × 10^–14^ × exp(390/*T*) cm^3^ molecule^–1^ s^–1^^6^ and *k*_4_(IUPAC) = 1.03 × 10^–13^ × exp((365 ± 200)/*T*) cm^3^ molecule^–1^ s^–1^.^5^ On average, the results of this work are ∼40% lower than the values calculated using the JPL and IUPAC recommendations. However, the results of this work have overlapping error limits at the 2σ level with both recommendations.

The previous studies upon which the JPL^6^ and IUPAC^5^ recommendations are based utilized the UV-absorption of CH_3_O_2_ at fixed wavelengths in the range ∼210–270 nm (typically 250 nm) usually to determine the ratio between the observed rate coefficient and the absorption cross-section of CH_3_O_2_, *k*_obs_/σ_CH3O2_.^5,6^ The values used for σ_CH3O2_ by the previous UV-absorption studies vary significantly, between (2.5–4.8) × 10^–18^ cm^2^ molecule^–1^ at 250 nm, leading to a large variation in *k*_obs_ across the studies, which at 298 K ranges between *k*_obs_ = (3.0–5.9) × 10^–13^ cm^3^ molecule^–1^ s^–1^.^7−14^ The 2020 JPL^6^ evaluation report recommends the cross sections obtained by the re-evaluation of Tyndall et al. in 2001^21^ of the previous reported UV-absorption spectra. At 250 nm Tyndall et al.^21^ recommend σ_CH3O2(250 nm)_ = 3.8 × 10^–18^ cm^2^ molecule^–1^. Our calculations show that using σ_CH3O2_ reported by Tyndall et al.^21^ and the ratios *k*_obs_/σ_CH3O2_ found in the previous kinetic studies,^7−11,13,14^ the range of the values of *k*_obs_ at 298 K is reduced to (4.1–5.1) × 10^–13^ cm^3^ molecule^–1^ s^–1^. The present result at 298 K, *k*_obs_ = 2.9 × 10^–13^ cm^3^ molecule^–1^ s^–1^, is 30% smaller than the lowest value of 4.1 × 10^–13^ cm^3^ molecule^–1^ s^–1^ and 40% lower than the JPL^6^ and IUPAC^5^ recommendations of *k*_obs_ = 4.8 × 10^–13^ cm^3^ molecule^–1^ s^–1^.

There is a significant difference between the results obtained here and the recommendations,^5,6^ and here we explore possible reasons for this discrepancy. The previous studies used either the flash photolysis (FP) technique^7−11^ or the molecular modulation (MM) method^12−14^ to generate CH_3_O_2_ radicals employing photolytic mixtures of CH_4_/Cl_2_/O_2_. The discrepancy between *k*_obs_ determined in here and the results reported in the FP studies^7−11^ could be due to unaccounted secondary chemistry of CH_3_O_2_ due to the high radical concentrations, on the order of [CH_3_O_2_] of 10^13^–10^14^ molecules cm^–3^, and/or unaccounted spectral interferences. The MM experiments^12−14^ used 1–2 orders of magnitude lower concentrations of CH_3_O_2_ than [CH_3_O_2_] in the FP studies,^7−11^ i.e., concentrations on the order 10^12^ molecules cm^–3^, to minimize the impact of the secondary chemistry on *k*_obs_. However, as this method consisted of modulating the photolysis and hence the production of radicals by alternating the time with the lamps switched on and the time with the lamps turned off, a potential important source of error was the buildup of products absorbing in the UV range of the measurements (see below). The contributions of the absorbing products (see below) were subtracted from the overall absorbance measured by the MM experiments^12,14^ to extract the absorbance of CH_3_O_2_, and thus the extracted absorbance depended on the concentrations and the cross sections attributed to the products. Note that the LIF method is selective and more sensitive, with a limit of detection for CH_3_O_2_ of 2.0 × 10^9^ molecules cm^–3^ for a signal-to-noise ratio of 2, 1 s averaging time of the online data points measured during the kinetic decay, and 60 s averaging period for the offline data points recorded at the end of the experiment. Therefore, the LIF method requires significantly lower radical concentrations than the FP and MM studies; here, [CH_3_O_2_]_0_ = (0.1–1) × 10^12^ molecules cm^–3^, which helps to minimize potential secondary chemistry.

The FP studies^7−11^ typically derived the *k*_obs_/σ_CH3O2_ ratio fitting either eq 10 or eq 11 to the measured optical absorbance (*A*_t_) or absorption coefficient (α_t_) at/around 250 nm.
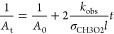
10
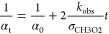
11Here *A*_0_ and α_0_ are the absorbance and the absorption coefficient at the time zero of the reaction, respectively, and *l* is the total optical path length.

To investigate the potential impact of the secondary chemistry on the CH_3_O_2_ kinetic decays in the FP studies^7−11^ numerical simulations were performed using a reaction system at 298 K described in the Supporting Information (Table S1). The previous kinetic studies of the CH_3_O_2_ recombination reaction^7−14^ did not investigate the impact of the CH_3_O_2_ reaction with the ClO radicals produced by the CH_3_O_2_ + Cl reaction on the CH_3_O_2_ kinetic decay. The kinetics of the CH_3_O_2_ + Cl reaction^34,35^ was studied in the years around 1995 and thus after all the previous kinetic studies of the CH_3_O_2_ self-reaction (1980–1990).^5,6^ The CH_3_O_2_ + Cl reaction is fast producing ClO (rate coefficient of 7.7 × 10^–11^ cm^3^ molecule^–1^ s^–1^ at 298 K).^34^ The generated ClO radicals predominantly react with CH_3_O_2_ with an overall rate coefficient of 2.4 × 10^–12^ cm^3^ molecule^–1^ s^–1^ at 298 K.^6^

The simulations used *k*_4_ = 2.1 × 10^–13^ cm^3^ molecule^–1^ s^–1^ as determined in this work and *r*_CH3O_ = 0.37.^5,6^ The Supporting Information shows examples of the results of the numerical simulations using concentrations representative for the FP studies:^7−11^ [CH_4_]_0_ = 5 × 10^17^ molecules cm^–3^ and [Cl]_0_ = 1.4 × 10^14^ molecules cm^–3^. As the Cl + CH_4_ reaction is relatively slow (rate coefficient = 1.0 × 10^–13^ cm^3^ molecule^–1^ s^–1^ at 298 K)^36^ about 7% of the Cl atoms react with CH_3_O_2_ to produce ClO. Subsequently ClO predominantly reacts with CH_3_O_2_ (peak [CH_3_O_2_] = 1.0 × 10^14^ molecules cm^–3^ as shown in the Supporting Information). Therefore, fitting eq 8 to the CH_3_O_2_ temporal decay simulated using the mechanism which includes the secondary chemistry results in *k*_obs_(fit) = (3.8 ± 0.1) × 10^–13^ cm^3^ molecule^–1^ s^–1^. Using eq 9, *k*_4_(fit) = 2.8 × 10^–13^ cm^3^ molecule^–1^ s^–1^ is obtained, which is 33% higher than the value of *k*_4_ determined from this work and used as an input in the numerical simulations, *k*_4_(simulations) = 2.1 × 10^–13^ cm^3^ molecule^–1^ s^–1^. To investigate the sensitivity of the results to the presence of the CH_3_O_2_ + ClO reaction in the chemical mechanism used, the simulations with [CH_4_]_0_ = 5 × 10^17^ molecules cm^–3^ and [Cl]_0_ = 1.4 × 10^14^ molecules cm^–3^ were repeated, but this time in the absence of the CH_3_O_2_ + ClO reaction. Without the CH_3_O_2_ + ClO reaction, *k*_obs_(fit) = (3.0 ± 0.1) × 10^–13^ cm^3^ molecule^–1^ s^–1^ resulting in *k*_4_(fit) = 2.2 × 10^–13^ cm^3^ molecule^–1^ s^–1^ being obtained, which is almost the same with the value used as input in the simulations, *k*_4_(simulations) = 2.1 × 10^–13^ cm^3^ molecule^–1^ s^–1^. Hence, for [CH_4_]_0_ = 5 × 10^17^ molecules cm^–3^ and [Cl]_0_ = 1.4 × 10^14^ molecules cm^–3^, the values of *k*_obs_ and *k*_4_ obtained from the fit to the simulated CH_3_O_2_ decay are considerably larger if the secondary chemistry is included to generate the decay. As the studies using the flash photolysis technique^7−11^ typically employed ∼[CH_4_]_0_ = 10^17^ molecules cm^–3^ and ∼[Cl]_0_ = 10^14^ molecules cm^–3^ the studies significantly overestimated *k*_obs_ and thus *k*_4_.

In the present study the concentrations of [CH_3_O_2_], [Cl], and [ClO] were in a steady-state with the lamps turned on, and the CH_3_O_2_ decays were generated by switching the lamps off. Numerical simulations were performed over 5 min to mimic the chemistry with the lamps turned on using [CH_4_]_0_ = 3.0 × 10^17^ molecules cm^–3^ with [Cl_2_]_0_ = 3.0 × 10^14^ molecules cm^–3^ representative for the present study and adding the Cl_2_ photolysis to the chemistry mechanism including the CH_3_O_2_ + Cl and CH_3_O_2_ + ClO reactions (Supporting Information). After 5 min with the lamps on, [CH_3_O_2_] = 5 × 10^11^ molecules cm^–3^, [Cl] = 7.0 × 10^6^ molecules cm^–3^, and [ClO] = 2.4 × 10^8^ molecules cm^–3^. The concentrations of all the species in the chemistry mechanism obtained after 5 min were input into numerical simulations using the same chemistry mechanism except without Cl_2_ photolysis to mimic the chemistry during the time with the lamps turned off. Virtually all Cl atoms and ClO radicals were removed on a time scale of hundreds of microseconds and seconds, respectively. As [Cl]_0_ = 7.0 × 10^6^ molecules cm^–3^ and [ClO]_0_ = 2.4 × 10^8^ molecules cm^–3^, and hence orders of magnitude lower than [Cl]_0_ = 1.4 × 10^14^ molecules cm^–3^ and a peak of [ClO] = 1.0 × 10^13^ molecules cm^–3^ in the simulations using concentrations representative for the FP studies,^7−11^ the simulated CH_3_O_2_ decays were not impacted by the chlorine species secondary chemistry (Figure S3). The fit of eq 9 to the CH_3_O_2_ decay provided a value of *k*_obs_(fit) = (3.0 ± 0.1) × 10^–13^ cm^3^ molecule^–1^ s^–1^ at 298 K, which results in *k*_4_(fit) = 2.2 × 10^–13^ cm^3^ molecule^–1^ s^–1^, i.e., practically the same as the value measured by the present experiments *k*_4_ = 2.1 × 10^–13^ cm^3^ molecule^–1^ s^–1^. Therefore, no impact by the secondary chemistry of CH_3_O_2_ included in the simulations (Supporting Information) on the value determined for *k*_4_ was found in the present study. Hence in the absence of secondary chemistry the value of *k*_4_, as determined in this work, is considerably lower than *k*_4_ when the secondary chemistry is present as a result of much higher initial [Cl] concentrations.

We now consider potential spectral interferences in previous work which monitored CH_3_O_2_ concentrations using UV absorption. At the typical λ = 250 nm used to monitor the CH_3_O_2_ absorption by the FP studies,^7−11^ the cross-section of ClO, σ_ClO_ = 3.5 × 10^–18^ cm^2^ molecule^–1^^6^ and the cross-section of CH_3_O_2_, σ_CH3O2_ = 3.8 × 10^–18^ cm^2^ molecule^–1^^21^ are similar, and hence numerical simulations were used to investigate the impact of any ClO spectral interference on the results of the FP studies. The concentrations of the species generated by numerical simulations using the representative concentrations for the FP studies of [CH_4_]_0_ = 5 × 10^17^ molecules cm^–3^ and [Cl]_0_ = 1.4 × 10^14^ molecules cm^–3^ in the model that includes the CH_3_O_2_ + ClO reaction were multiplied with their respective cross sections^6,21^ to obtain the absorption coefficients of CH_3_O_2_ and ClO:
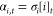
12where α_*i*, *t*_ is the absorption coefficient of species *i* (CH_3_O_2_ or ClO) at reaction time *t*, *σ*_*i*_ is the absorption cross-section of species *i* at 250 nm and [*i*]_*t*_ is the concentration of species *i* at time *t*. Figure S4 in the Supporting Information shows the generated α_CH3O2, *t*_ and α_ClO, *t*_ and their sum, Σα_*i*, *t*_ = α_CH3O2, *t*_*+ α*_ClO, *t*_. The results (Figure S4) show that there is a minor contribution of α_ClO, *t*_ to Σα_*i*, *t*_ at the start of the reaction, i.e., ∼8% in the first millisecond of the reaction, which drops to 3% at *t* = 10 ms. The fit of the eq 11 (see above) to Σα_*i*, *t*_ vs time resulted in *k*_obs_/σ_CH3O2_. Using σ_CH3O2_ = 3.8 × 10^–18^ cm^2^ molecule^–1^^21^ a value of *k*_obs_ = (3.9 ± 0.1) × 10^–13^ cm^3^ molecule^–1^ s^–1^ is obtained, which is 3% higher than *k*_obs_ given fitting eq 9 to the temporal decay of [CH_3_O_2_] generated by the same numerical simulations (Figure S1). The result suggests that there was no significant optical interference due to the ClO absorption which impacted the previous determinations of *k*_obs_ using the FP technique.^7−11^

The molecular modulation (MM) studies^12−14^ used the time-resolved modulated UV-absorption (absorption waveform) generated in the range 210–270 nm via switching the photolysis lamps on and off with a typical frequency of 10^–1^ Hz (order of magnitude) to determine *k*_obs_ and σ_CH3O2_. The absorption waveform consisted of an initial rise followed by a pseudo-steady-state and then a decay during the dark phase of the modulation period.^12−14^ The modulated absorption components depended on a relatively large number of parameters: illumination time, rate of Cl_2_ photolysis, kinetic parameters, and absorbing species cross sections.^12−14^ Photolytic mixtures of CH_4_/Cl_2_/O_2_ were flowed through the reactor to minimize the buildup of the products. However, the residence times of the gases in the reaction cell were relatively long, for example, 35 and 60 s.^13,14^ The contributions of the absorbing products accumulating over the photolysis cycles was calculated and subtracted from the observed absorption to derive the modulated absorption of CH_3_O_2_.^12,14^ The contribution of the products were important in the range 210–250 nm where the cross sections of HO_2_ (formed by reaction R2 and the reactions of Cl with the products CH_3_OH and CH_2_O) and CH_3_OOH (produced by the CH_3_O_2_ + HO_2_ reaction) increases rapidly with decreasing λ.^6^ However, the cross sections of HO_2_ and/or CH_3_OOH were significantly overestimated in the MM studies.^12−14^ The references cited for the cross-section of HO_2_ by Cox and Tyndall^12^ reported σ_HO2_ larger than the JPL recommendations^6^ by 60–70% in the range 210–250 nm^37^ and by ∼10% between 210–220 nm.^38^ The cross-section of HO_2_^39^ used by Simon et al.^14^ is ∼30% larger than the JPL recommendation^6^ between 210–240 nm and by ∼60% at 250 nm. Both the studies of Cox and Tyndall^12^ and Jenkin et al.^13^ employed values for σ_CH3OOH_ at least 40–50% higher than the JPL recommendation in the range 210–250 nm.^6^ An overestimation of the contributions of these species to the measured absorbance results in an underestimation of the CH_3_O_2_ absorbance, *A*_CH3O2_, which in turn results in an overestimation of *k*_obs_/σ_CH3O2_ (eq 10).

In addition, the CH_3_O_2_ loss to the walls of the reaction cell were not accounted for by neither the FP studies^7−11^ nor the MM studies^12−14^ and could also result in an overestimation of *k*_obs_. As the CH_3_O_2_ self-reaction is slow, the wall-loss could significantly contribute to the overall CH_3_O_2_ removal in the previous studies.

## Discussion of the FAGE Instrument Calibration

As LIF is not an absolute detection method, the FAGE instrument required calibration and a calibration factor, *C*_CH_3_O_2__, is used to convert the measured signal, *S*_CH_3_O_2__, to the CH_3_O_2_ concentration:
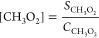
5To calibrate FAGE, CH_3_O_2_ radicals were generated in known concentrations employing the 184.9 nm photolysis of water vapor in synthetic air followed by the complete conversion of the generated OH radicals to CH_3_O_2_ by reaction with CH_4_ in a large excess in the presence of O_2_. Previous work described in detail the water vapor method of calibration and the uncertainties in the calibration factor, *C*_CH_3_O_2__(water vapor method).^22^ As seen previously,^22^ in this work an overall 34% error at 2σ level was obtained for *C*_CH_3_O_2__(water vapor method) combining the systematic and statistical uncertainties. Similar overall errors, 31% and 36%, were reported for *C*_HO_2__(water vapor method) previously.^28,30^

The photolysis of water vapor at 184.9 nm represents the most common method used to generate accurate concentrations of OH and HO_2_. The method has been applied for many years for the calibration of FAGE instruments.^24−26^ The reliability of the method has been confirmed by intercomparisons with alternative methods of calibration for OH and HO_2_. The calibration of OH using the water vapor photolysis and the OH calibration based on the generation of OH by ozone reactions with alkenes have been found to agree within their experimental uncertainties.^40^ Very good agreement (difference within 1–13%) has been obtained by comparing the OH measurements in the SAPHIR atmospheric simulation chamber using a number of FAGE instruments and instruments employing differential optical laser absorption spectroscopy (DOAS) and chemical ionization mass spectrometry (CIMS).^41,42^ In the case of HO_2_, *C*_HO_2__(water vapor method) and the calibration factor obtained analyzing the kinetic decay of HO_2_ by its self-reaction generated in HIRAC, *C*_HO_2__(kinetic method), were found in a very good agreement (difference within 8%).^28,30^ However, a discrepancy within ∼40% was found between *C*_CH_3_O_2__(water vapor method) and the CH_3_O_2_ calibration factor determined using the kinetics of the second-order recombination of CH_3_O_2_ observed in HIRAC and *k*_obs_(298 K) = 4.8 × 10^–13^ cm^3^ molecule^–1^ s^–1^,^5,6^*C*_CH_3_O_2__(kinetic method).^22,23^ As the error in the fraction of OH which is converted to CH_3_O_2_ upon the addition of methane in the water vapor method is minor (4% at 2σ level),^22^ the discrepancy between the two calibration methods can be attributed to an overestimation of the reported value of *k*_obs_ for the CH_3_O_2_ self-reaction at 298 K.^5,6^ The ∼40% difference in *C*_CH_3_O_2__(kinetic method) and *C*_CH_3_O_2__(water vapor method) resulted in different values for the gradient of the correlation plot of [CH_3_O_2_] measured by FAGE (*y*-axis) as a function of [CH_3_O_2_] measured by near-infrared cavity ring down spectroscopy, CRDS (*x*-axis), at 1000 mbar of synthetic air using the sensitivities from the two methods of calibration of FAGE: 1.35 ± 0.07 (water vapor calibration) and 0.92 ± 0.05 (kinetic method of calibration).^23^ The results show a significantly better agreement with the kinetic method than with the water vapor method. A very good level of agreement between the FAGE and CRDS measurements of CH_3_O_2_ was also obtained using the kinetic method for FAGE calibration at 100 mbar of synthetic air and 80 mbar of 3:1 He:O_2_ mixture. The very good agreement achieved under all conditions when the kinetic method was employed for the FAGE calibration was expected as the kinetic method was also used to determine the absorption cross section of CH_3_O_2_ from the temporal decays of the optical absorption coefficient of CH_3_O_2_ and hence calibrate the CRDS method. Therefore, with both FAGE and CRDS calibrated using the same method, the intercomparison was not subject to any error in the rate coefficient, *k*_obs_ for the CH_3_O_2_ self-reaction, and the obtained very good agreement provides a validation of the FAGE (water vapor) method to determine concentrations of CH_3_O_2_. The present result at 298 K, *k*_obs_ = 2.9 × 10^–13^ cm^3^ molecule^–1^ s^–1^, shows a 40% reduction in the reported value of *k*_obs_ = 4.8 × 10^–13^ cm^3^ molecule^–1^ s^–1^.^5,6^ A reduction of 40% in the reported *k*_obs_ would bring [CH_3_O_2_]_CRDS_ generated using the kinetic method of calibration into agreement with [CH_3_O_2_]_FAGE_ determined using the water vapor method.^23^ Therefore, the FAGE–CRDS intercomparison also suggests that *k*_obs_ = 2.9 × 10^–13^ cm^3^ molecule^–1^ s^–1^ and thus *k*_4_ = 2.1 × 10^–13^ cm^3^ molecule^–1^ s^–1^ at 298 K, consistent with the values found in the present study.

## Conclusions

Experiments were carried out in the range 268–344 K and at 1000 mbar to measure CH_3_OH and CH_2_O generated by the CH_3_O_2_ self-reaction in HIRAC using *in situ* FTIR detection to determine the product branching ratios in the self-reaction. The chemistry was initiated using photolysis of Cl_2_/CH_4_/N_2_/O_2_ mixtures (photolysis range: λ = 350–400 nm). The temperature dependence of the product branching ratio of the reaction channel producing CH_3_O can be described as *r*_CH3O_ = 1/{1 + [exp(600 ± 170)/*T*]/(3.9 ± 2.2)}. At 295 K *r*_CH3O(this work)_ = 0.34 ± 0.05, in agreement with the recommendations of JPL^6^ and IUPAC:^5^*r*_CH3O(JPL)_ = 0.36 ± 0.12 and *r*_CH3O(IUPAC)_ = 0.36 ± 0.17. This is the second experimental study of the temperature dependence of the product branching ratios in a range including temperatures relevant for atmospheric chemistry. The positive temperature dependence found for *r*_CH3O_ is less marked than the increase in *r*_CH3O_ with temperature measured by the previous study performed in a temperature range around 298 K (223–333 K), which used matrix isolation FTIR.^20^ The results of the present work for product branching agree well with the values obtained by Horie et al.^20^ at 295, 323, and 344 K. By decreasing the temperature, the present results are increasingly higher than the results reported by Horie et al.^20^ with a positive deviation of 22% at 268 K.

The kinetics of the CH_3_O_2_ self-reaction has been studied coupling a FAGE instrument to HIRAC to carry out time-resolved measurements of the CH_3_O_2_ concentrations during the reaction. Second-order decays of CH_3_O_2_ were generated by turning the chamber lamps off. The observed rate coefficient at 295 K and 1000 mbar was *k*_obs_ = (2.7 ± 0.9) × 10^–13^ cm^3^ molecule^–1^ s^–1^. Using *k*_obs_(295 K) = *k*_4_(1 + *r*_CH3O_) with *r*_CH3O(this work, 295 K)_ = 0.34 ± 0.05 the second-order rate coefficient for the self-reaction at 295 K and 1000 mbar is *k*_4_ = (2.0 ± 0.7) × 10^–13^ cm^3^ molecule^–1^ s^–1^; employing the recommended value of 0.36 for *r*_CH3O_ at 295 K by JPL^6^ and IUPAC^5^ does not change the result. The result at 295 K is is ∼40% lower than the IUPAC and JPL recommendations: *k*_4_ = 3.5 × 10^–13^ cm^3^ molecule^–1^ s^–1^. The temperature dependence of the overall rate coefficient can be parametrized as *k*_4_ = (9.1 ± 5.3) × 10^–14^ × exp((252 ± 174)/*T*) cm^3^ molecule^–1^ s^–1^. The present results have overlapping error limits at the 2σ level with both JPL and IUPAC recommendations. However, on average the results of this work are ∼40% lower than the values calculated using the JPL^6^ recommendation for the temperature dependence (*k*_4_ = 9.5 × 10^–14^ × exp(390/*T*) cm^3^ molecule^–1^ s^–1^) and the values obtained employing the temperature dependence recommended by IUPAC,^5^*k*_4_ = 1.03 × 10^–13^ × exp((365 ± 200)/*T*) cm^3^ molecule^–1^ s^–1^.

The previous kinetic studies utilized UV–absorption spectroscopy and may be impacted by secondary chemistry owing to the high radical concentrations generated in the reaction mixtures. Chemical modeling using the conditions of the previous studies and which included secondary chemistry of Cl species showed that the secondary chemistry increases the value of *k*_4_ obtained significantly. The FAGE method detects CH_3_O_2_ sensitively, with a limit of detection of 2.0 × 10^9^ molecules cm^–3^ for a signal-to-noise ratio of 2, 1 s online averaging time, and 60 s offline averaging period. Therefore, the experiments reported here required a few orders of magnitude lower concentrations of CH_3_O_2_ than [CH_3_O_2_] used in the UV–absorption studies and the impact of the secondary chemistry on the kinetic decays obtained by this work is negligible. In addition, the FAGE method probes CH_3_O_2_ selectively, in the absence of any interference from other species.

Numerical models predict that CH_3_O_2_ is the most abundant RO_2_ species in the atmosphere. Even though CH_3_O_2_ has not been selectively measured in the atmosphere so far, its concentration at daytime has been estimated to peak in the range of daytime peak [HO_2_], i.e., at 10^7^–10^8^ molecules cm^–3^.^43−45^ The atmospheric fate of CH_3_O_2_ is typically dominated by the reaction with NO, with the CH_3_O_2_ + HO_2_ reaction becoming the main daytime loss of CH_3_O_2_ under low NO_*x*_ levels. As CH_3_O_2_ and HO_2_ reach similar levels at daytime and 298K, *k*_CH3O2+HO2_ (5.2 × 10^–12^ cm^3^ molecule^–1^ s^–1^) is ∼15 times faster than the JPL and IUPAC recommendations for *k*_4_ (3.5 × 10^–13^ cm^3^ molecule^–1^ s^–1^)^5,6^ and ∼25 times higher than *k*_4(CH3O2+CH3O2)_ determined in this work (2.1 × 10^–13^ cm^3^ molecule^–1^ s^–1^) the inclusion of the present *k*_obs(CH3O2+CH3O2)_ value in the atmospheric models might not impact significantly the daytime radical budget predicted by the models. However, at night-time it has been predicted that the self-reaction is the dominant removal of CH_3_O_2_ due to a rapid loss of HO_2_ under dark conditions^46^ and thus the atmospheric impacts of the present result need to be investigated.

## Supporting Information Available

Results of numerical simulations using a complex chemistry system and details of determination of the branching ratios in the CH_3_O_2_ self-reaction as a function of temperature (PDF)
