# Supplementary material for: Kinetics and Product Branching Ratio Study of the CH3O2 Self-Reaction in the Highly Instrumented Reactor for Atmospheric Chemistry
Source: J Phys Chem A. 2022 Oct 13;126(42):7639–49. doi: 10.1021/acs.jpca.2c04968 (PMC9620170; doi:10.1021/acs.jpca.2c04968)
Supplement: Supplementary file 1 — jp2c04968_si_001.pdf [file jp2c04968_si_001.pdf]

# Supporting Information

# Kinetics and Product Branching Ratio Study of the $CH_3O_2$ Self-Reaction in the Highly Instrumented Reactor for Atmospheric Chemistry

Lavinia Onel[1], Alexander Brennan[1], Freja F. Østerstrøm[1†], Ellie Cooke[1], Lisa Whalley[1,2], Paul W. Seakins[1], Dwayne E. Heard[1*]

[1]School of Chemistry, University of Leeds, Leeds, LS2 9JT, UK
[2]National Centre for Atmospheric Science, University of Leeds, LS2 9JT, UK
[†] Now at: School of Engineering and Applied Sciences, Harvard University, Cambridge, MA 02138, USA & Department of Chemistry, University of Copenhagen, 2100 Copenhagen Ø, Denmark

* *Correspondence to*: d.e.heard@leeds.ac.uk; +44 113 343 6471

## S1. Results of numerical simulations using a complex chemistry system

Numerical simulations were performed using the reactions shown in Table S1 to investigate the discrepancy between the value of the overall rate coefficient of the $CH_3O_2$ self-reaction ($k_4$) obtained in this work and the value of $k_4$ determined using the flash photolysis (FP)–UV absorption spectroscopy previously.[1-5] The impact of the $CH_3O_2$ + Cl reaction followed by the reaction of $CH_3O_2$ with ClO on $k_4$ in the FP studies was investigated.

**Table S1**. Reactions and rate coefficients used in numerical simulations

| **Reaction** | **Rate coefficient at 298 K / $cm^3$ $molecule^{-1}$ $s^{-1}$ or $s^{-1}$** | **Reference** |
|---|---|---|
| $CH_4 + Cl \rightarrow CH_3 + HCl$ | $1.0 \times 10^{-13}$ | Atkinson et al.[6] |
| $CH_3 + O_2 \rightarrow CH_3O_2$ | $9.5 \times 10^{-13}$ | Atkinson et al.[6] |
| $CH_3O_2 + CH_3O_2 \rightarrow CH_3OH + CH_2O + O_2$ | $1.3 \times 10^{-13}$ | this work |
| $CH_3O_2 + CH_3O_2 \rightarrow CH_3O + CH_3O + O_2$ | $7.8 \times 10^{-14}$ | this work |
| $CH_3O + O_2 \rightarrow CH_2O + HO_2$ | $1.9 \times 10^{-15}$ | Atkinson et al.[6] |
| $CH_3O_2 + HO_2 \rightarrow CH_3OOH + O_2$ | $4.7\times 10^{-12}$ | Atkinson et al.[6] |
| $CH_3O_2 + HO_2 \rightarrow CH_2O + O_2 + H_2O$ | $5.2 \times 10^{-13}$ | Atkinson et al.[6] |

| | | |
|---|---|---|
| $HO_2 + HO_2 \rightarrow H_2O_2 + O_2$ | $2.9 \times 10^{-12}$ | Atkinson et al.[6] |
| $CH_3OH + Cl \rightarrow CH_2OH + HCl$ | $5.5 \times 10^{-11}$ | Atkinson et al.[6] |
| $CH_2OH + O_2 \rightarrow CH_2O + HO_2$ | $9.7 \times 10^{-12}$ | Atkinson et al.[6] |
| $CH_2O + Cl + O_2 \rightarrow CO + HO_2 + HCl$ | $7.2 \times 10^{-11}$ | Atkinson et al.[6] |
| $CH_3OOH + Cl \rightarrow$ Products | $5.9 \times 10^{-11}$ | Atkinson et al.[6] |
| $H_2O_2 + Cl \rightarrow HO_2 + HCl$ | $4.1 \times 10^{-13}$ | Atkinson et al.[6] |
| $CH_3 + Cl \rightarrow CH_3Cl$ | $6.1 \times 10^{-11}$ | Parker et al.[7] |
| $CH_3 + Cl_2 \rightarrow CH_3Cl + Cl$ | $1.6 \times 10^{-12}$ | Eskola et al.[8] |
| $CH_3O_2 + Cl \rightarrow CH_3O + ClO$ | $7.7 \times 10^{-11}$ | Maricq et al.[9] |
| $CH_3O_2 + Cl \rightarrow CH_2OO + HCl$ | $7.4 \times 10^{-11}$ | Maricq et al.[9] |
| $HO_2 + Cl \rightarrow O_2 + HCl$ | $3.4 \times 10^{-11}$ | Atkinson et al.[10] |
| $HO_2 + Cl \rightarrow ClO + OH$ | $9.3 \times 10^{-12}$ | Atkinson et al.[10] |
| $HO_2 + ClO \rightarrow$ Products | $6.9 \times 10^{-12}$ | Atkinson et al.[10] |
| $CH_3O_2 + ClO \rightarrow$ Products | $2.4 \times 10^{-12}$ | Burkholder et al.[11] |
| $ClO + ClO \rightarrow Cl_2 + O_2$ | $4.8 \times 10^{-15}$ | Atkinson et al.[10] |
| $ClO + ClO \rightarrow Cl + ClO_2$ | $8.0 \times 10^{-15}$ | Atkinson et al.[10] |
| $ClO + ClO \rightarrow Cl + OClO$ | $3.5 \times 10^{-15}$ | Atkinson et al.[10] |
| $CH_3 + CH_3O_2 \rightarrow CH_3O + CH_3O$ | $4.5 \times 10^{-11}$ | Pilling et al.[12] |
| $CH_3O + CH_3O_2 \rightarrow$ Products | $2.6 \times 10^{-12}$ | Heicklen[13] |
| $CH_3O + CH_3O \rightarrow CH_2O + CH_3OH$ | $3.9 \times 10^{-11}$ | Hassinen and Koskikallio[14] |
| $HO_2 + CH_2O \rightarrow HOCH_2O_2$ | $7.9 \times 10^{-14}$ | Atkinson et al.[6] |
| $HOCH_2O_2 \rightarrow HO_2 + CH_2O$ | $1.5 \times 10^{2}$ | Atkinson et al.[6] |
| $HO_2 + HOCH_2O_2 \rightarrow HOCH_2OOH + O_2$ | $6.0 \times 10^{-12}$ | Atkinson et al.[6] |
| $HO_2 + HOCH_2O_2 \rightarrow HCOOH + H_2O + O_2$ | $3.6 \times 10^{-12}$ | Atkinson et al.[6] |
| $HO_2 + HOCH_2O_2 \rightarrow OH + HOCH_2O + O_2$ | $2.4 \times 10^{-12}$ | Atkinson et al.[6] |
| $HOCH_2O_2 + HOCH_2O_2 \rightarrow HCOOH + HOCH_2OH + O_2$ | $7.0 \times 10^{-13}$ | Atkinson et al.[6] |
| $HOCH_2O_2 + HOCH_2O_2 \rightarrow HOCH_2O + HOCH_2O + O_2$ | $5.5\times 10^{-12}$ | Atkinson et al.[6] |

Simulations employed the value obtained at 298 K in this work for the overall rate coefficient of the $CH_3O_2$ self-reaction, $k_4$(simulations) = 2.1 × $10^{-13}$ $cm^3$ $molecule^{-1}$ $s^{-1}$ and the branching ratio of the channel producing $CH_3O$ $r_{CH3O}$ = 0.37.[6, 11] Using $[CH_4]_0$ = 5 × $10^{17}$ molecule $cm^{-3}$ and $[Cl]_0$ = 1.4 × $10^{14}$ molecule $cm^{-3}$ the concentration time profiles for $CH_3O_2$, ClO and Cl shown in Figure S1 were generated. The initial concentration of Cl was chosen to generate a peak $[CH_3O_2]$ of 1.0 × $10^{14}$ molecule $cm^{-3}$, representative for the FP studies, that used $[CH_3O_2]$ of $10^{13}$ – $10^{14}$ molecule $cm^{-3}$ orders of magnitude. Figure S1 shows that Cl atoms are almost instantaneously removed, predominantly through the reaction with $CH_4$. However, 7% of the Cl atoms produces ClO by the reaction with $CH_3O_2$ and the main loss of ClO is by the reaction with $CH_3O_2$. The $CH_3O_2$ kinetic decays generated by numerical simulations were fitted using equation S1 (equation 9 in the main text) to determine $k_{obs}$.

$$\frac{1}{[\mathrm{CH_3O_2}]_t} = \frac{1}{[\mathrm{CH_3O_2}]_0} + 2\,k_{\mathrm{obs}}t. \tag{S1}$$

Here $[CH_3O_2]_t$ is the concentration of $CH_3O_2$ at time $t$ and $[CH_3O_2]_0$ is the concentration of $CH_3O_2$ at $t$ = 0. The fit shown in Figure S1 resulted in $k_{obs}$ = (3.8 ± 0.1) × $10^{-13}$ $cm^3$ $molecule^{-1}$ $s^{-1}$. Then the rate coefficient of the $CH_3O_2$ self-reaction, $k_4$ was derived from $k_{obs}$ using equation S2 (equation 1 in the main text) with $r_{CH3O}$ = 0.37 to determine $k_4$(fit) = 2.8 × $10^{-13}$ $cm^3$ $molecule^{-1}$ $s^{-1}$. This value is 33% higher than $k_4$(simulations) = 2.1 × $10^{-13}$ $cm^3$ $molecule^{-1}$ $s^{-1}$.

$$k_{\mathrm{obs}} = k_4(1 + r_{\mathrm{CH3O}}), \tag{S2}$$

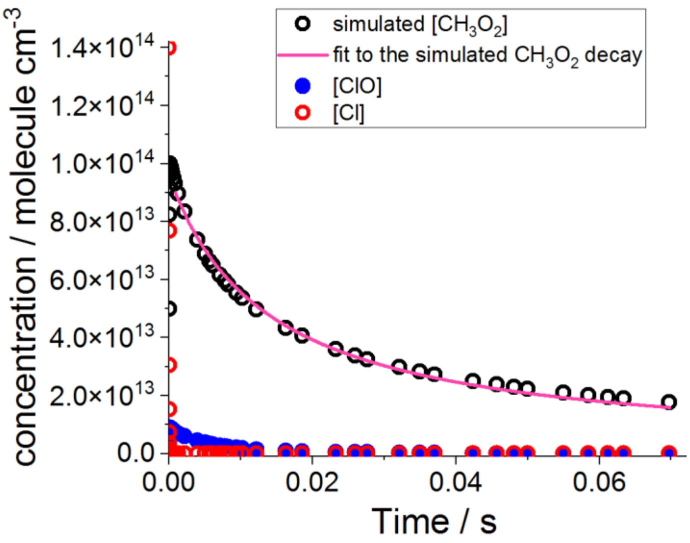


**Figure S1**. Concentrations of $CH_3O_2$ (black), ClO (blue) and Cl (red) generated by numerical simulations employing $[Cl]_0 = 1.4 \times 10^{14}$ molecule $cm^{-3}$, $[CH_4]_0 = 5 \times 10^{17}$ molecule $cm^{-3}$ and $[O_2]_0 = 5 \times 10^{18}$ molecule $cm^{-3}$ and the reactions and rate coefficients at 298 K shown in Table S1. The fit of equation S1 (pink line) to the simulated $CH_3O_2$ temporal decay results in $k_{obs} = (3.8 \pm 0.1) \times 10^{-13}$ $cm^3$ molecule$^{-1}$ $s^{-1}$ that gives an overall rate coefficient of the $CH_3O_2$ self-reaction $k_4$(fit) = $2.8 \times 10^{-13}$ $cm^3$ molecule$^{-1}$ $s^{-1}$ by using equation S2. This value is 33% larger than the value of $k_4$(simulations) = $2.1 \times 10^{-13}$ $cm^3$ molecule$^{-1}$ $s^{-1}$ used for $k_4$ in the numerical simulations.

The simulations with $[CH_4]_0 = 5 \times 10^{17}$ molecule $cm^{-3}$ and $[Cl]_0 = 1.4 \times 10^{14}$ molecule $cm^{-3}$ were repeated, but this time in the absence of the $CH_3O_2$ + ClO reaction. The generated $CH_3O_2$ kinetic decays were fitted using equation S1 to obtain $k_{obs}$(fit) = $(3.0 \pm 0.1) \times 10^{-13}$ $cm^3$ molecule$^{-1}$ $s^{-1}$, which then was used in equation S2 to derive $k_4$(fit) = $2.2 \times 10^{-13}$ $cm^3$ molecule$^{-1}$ $s^{-1}$. This value is almost the same with the value used as input in the simulations, $k_4$(simulations) = $2.1 \times 10^{-13}$ $cm^3$ molecule$^{-1}$ $s^{-1}$. The result shows that $k_4$ obtained from the fit to the simulated $CH_3O_2$ decay is significantly overestimated if the $CH_3O_2$ + ClO reaction is included to generate the decay. As the FP studies typically employed $\sim[CH_4]_0 = 10^{17}$ molecule $cm^{-3}$ with high initial Cl concentrations of $\sim[Cl]_0 = 10^{14}$ molecule $cm^{-3}$, the unaccounted $CH_3O_2$ secondary chemistry significantly impacted the previous results.[1-5]

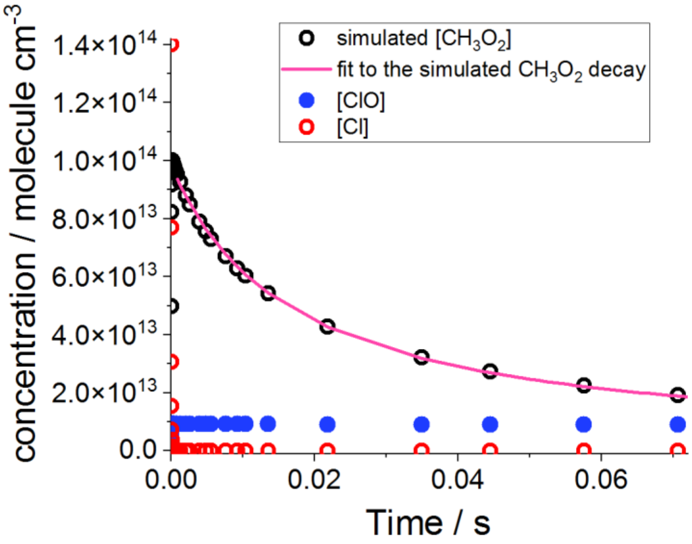


**Figure S2**. Concentrations of $CH_3O_2$ (black), ClO (blue) and Cl (red) generated by numerical simulations employing $[Cl]_0 = 1.4 \times 10^{14}$ molecule cm$^{-3}$, $[CH_4]_0 = 5 \times 10^{17}$ molecule cm$^{-3}$ and $[O_2]_0 = 5 \times 10^{18}$ molecule cm$^{-3}$ and the chemical mechanism described in Table S1 excluding the $CH_3O_2$ + ClO reaction. Fit of equation S1 (pink line) to the simulated $CH_3O_2$ temporal decay results in $k_{obs} = (3.0 \pm 0.1) \times 10^{-13}$ cm$^3$ molecule$^{-1}$ s$^{-1}$ that gives an overall rate coefficient of the $CH_3O_2$ self-reaction $k_4$(fit) = $2.2 \times 10^{-13}$ cm$^3$ molecule$^{-1}$ s$^{-1}$ by using equation S2. The value is practically the same as $k_4$(simulations) = 2.1 × 10-$^{-13}$ cm$^3$ molecule$^{-1}$ s$^{-1}$.

Numerical simulations were then used to investigate if there was any impact of the secondary reactions on the results obtained under the conditions of the present work. In this study the concentrations of $[CH_3O_2]$, [Cl] and [ClO] were in a steady-state with the lamps turned on and the $CH_3O_2$ decays were generated by switching the lamps off. The chemistry during the time with the lamps switched on was mimicked by adding $Cl_2$ photolysis (R6, reaction labelled as in main text) to the model described in Table S1, and including the $CH_3O_2$ + Cl and $CH_3O_2$ + ClO reactions.

$$Cl_2 + h\nu \rightarrow 2Cl, \qquad \text{(R6)}$$

with $j(Cl_2) = (3.8 \pm 1.1) \times 10^{-4}$ s$^{-1}$.[15, 16] Simulations using $[CH_4]_0 = 3.0 \times 10^{17}$ molecule cm$^{-3}$ and $[Cl_2]_0 = 3.0 \times 10^{14}$ molecule cm$^{-3}$, representative for the present work work, were performed for a duration of 5 min and generated $[CH_3O_2] = 5 \times 10^{11}$ molecule cm$^{-3}$, [Cl] = $7.0 \times 10^6$ molecule cm$^{-3}$ and [ClO] = $2.4 \times 10^8$ molecule cm$^{-3}$ at the end of the run. Therefore, the concentrations of Cl and ClO were orders of magnitude lower than $[Cl]_0 = 1.4 \times 10^{14}$ molecule cm$^{-3}$ and the peak of [ClO] = $1.0 \times 10^{13}$ molecule cm$^{-3}$ in the simulations using

concentrations representative for the FP studies.[1-5] The concentrations of all species in the chemical mechanism obtained after 5 min were input into simulations employing the same chemistry mechanism (Table S1) in the absence of the $Cl_2$ photolysis to mimic the chemistry with the lamps turned off. Simulations showed that Cl atoms and ClO radicals (Figure S3) were removed on a scale of hundreds of microseconds and seconds, respectively.

The fit of equation S1 to the $CH_3O_2$ decay (Figure S3) resulted in $k_{obs}$(fit) = (3.0 ± 0.1) × $10^{-13}$ $cm^3$ $molecule^{-1}$ $s^{-1}$, which provided $k_4$(fit) = 2.2 × $10^{-13}$ $cm^3$ $molecule^{-1}$ $s^{-1}$. This value is practically the same as the one determined by the present experiments $k_4$ = 2.1 × $10^{-13}$ $cm^3$ $molecule^{-1}$ $s^{-1}$. Therefore, negligible impact by the secondary chemistry of $CH_3O_2$ included in the simulations on the value obtained for $k_4$ was found in the present work.

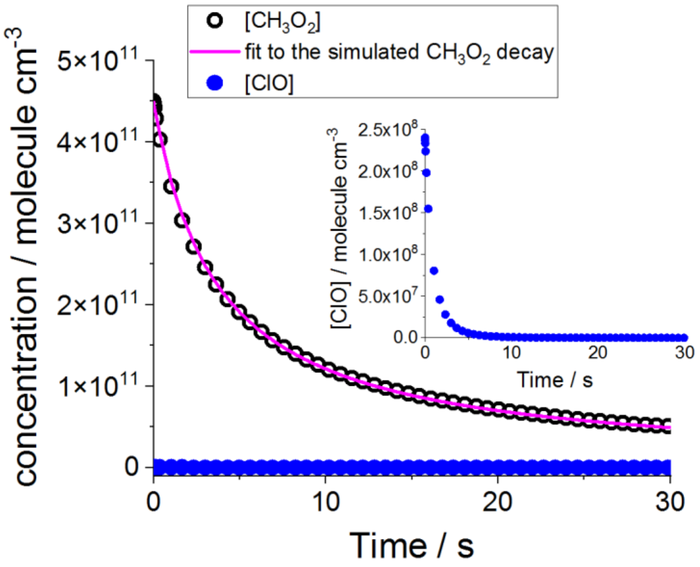


**Figure S3**. Concentrations of $CH_3O_2$ (black) and ClO (blue) generated by numerical simulations employing $[Cl]_0$ = 7.0 × $10^6$ molecule $cm^{-3}$, $[ClO]_0$ = 2.4 × $10^8$ molecule $cm^{-3}$, $[CH_4]_0$ = 3 × $10^{17}$ molecule $cm^{-3}$, $[O_2]_0$ = 5 × $10^{18}$ molecule $cm^{-3}$ and the chemical mechanism described in Table S1 including the $CH_3O_2$ + ClO reaction. Virtually all Cl atoms and ClO radicals were removed on a scale of hundred of microseconds and seconds, respectively. A fit of equation S1 (pink line) to the simulated $CH_3O_2$ temporal decay results in $k_{obs}$ = (3.0 ± 0.1) × $10^{-13}$ $cm^3$ $molecule^{-1}$ $s^{-1}$, that then gives practically the same value for the overall rate coefficient of the $CH_3O_2$ self-reaction $k_4$(fit) = 2.2 × $10^{-13}$ $cm^3$ $molecule^{-1}$ $s^{-1}$ as $k_4$(simulations) = 2.1 × $10^{-13}$ $cm^3$ $molecule^{-1}$ $s^{-1}$.

The FP studies typically measured the optical absorption of $CH_3O_2$ at $\lambda$ = 250 nm, where the cross-section of ClO $\sigma_{ClO}$ = 3.5 × $10^{-18}$ $cm^2$ $molecule^{-1}$ and the cross-section of $CH_3O_2$ $\sigma_{CH3O2}$ = 3.8 × $10^{-18}$ $cm^2$ $molecule^{-1}$ are similar. Therefore, simulations were carried out to understand if any spectral interference due to ClO impacted on the results of the FP studies. Absorption coefficients of $CH_3O_2$ and ClO were computed by multiplying $[CH_3O_2]$

and [ClO] generated by numerical simulations using $[CH_4]_0$ = 5 × $10^{17}$ molecule $cm^{-3}$ and $[Cl]_0$ = 1.4 × $10^{14}$ molecule $cm^{-3}$ in the model that includes the $CH_3O_2$ + ClO reaction (Table S1) with their respective cross-sections at 250 nm (equation S3, equation 12 in the main text).

$$\alpha_{i,\,t} = \sigma_i\,[i]_t, \qquad \text{(S3)}$$

where $\alpha_{i,\,t}$ is the absorption coefficient of species $i$ ($CH_3O_2$ or ClO) at reaction time $t$, $\sigma_i$ is the absorption cross-section of species $i$ at 250 nm and $[i]_t$ is the concentration of species $i$ at time $t$. The generated $\alpha_{CH3O2,\,t}$ and $\alpha_{ClO,\,t}$ and their sum, $\Sigma\alpha_{i,\,t} = \alpha_{CH3O2,\,t} + \alpha_{ClO,\,t}$ are shown in Figure S4. Figure S4 shows that the contribution of $\alpha_{ClO,\,t}$ to $\Sigma\alpha_{i,\,t}$ is minor. The temporal decay of $\Sigma\alpha_{i,\,t}$ was fitted by equation 11 in the main text to extract $k_{obs}/\sigma_{CH3O2}$ and then $k_{obs}$ = (3.9 ± 0.1) × $10^{-13}$ $cm^3$ $molecule^{-1}$ $s^{-1}$ using $\sigma_{CH3O2}$ = 3.8 × $10^{-18}$ $cm^2$ $molecule^{-1}$.[17] The value obtained for $k_{obs}$ is 3% higher than $k_{obs}$ determined fitting equation S1 to $[CH_3O_2]$ vs. time generated by the same numerical simulations (Figure S1). The result suggests that no significant optical interference due to ClO impacted the kinetic results reported by the FP studies.

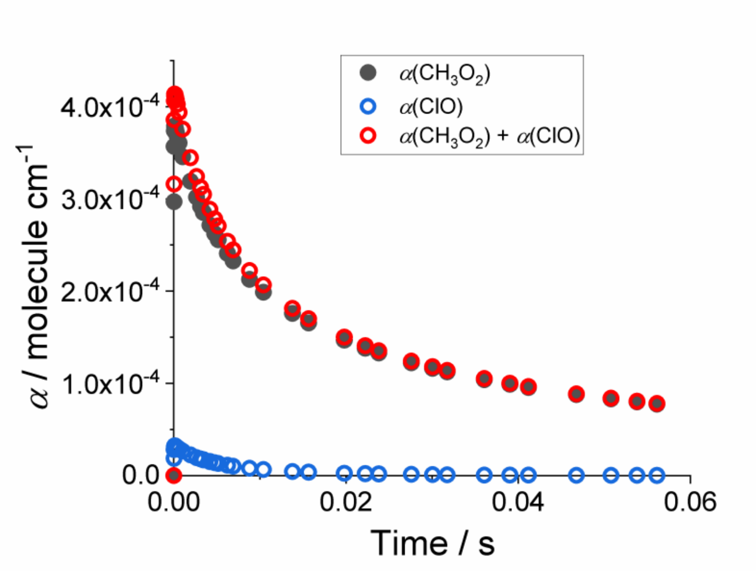


**Figure S4**. Time profiles of the absorption coefficients ($\alpha$) of $CH_3O_2$ (black), ClO (blue) and their sum (red). The absorption coefficients were generated by performing numerical simulations using $[Cl]_0$ = 1.4 × $10^{14}$ molecule $cm^{-3}$, $[CH_4]_0$ = 5 × $10^{17}$ molecule $cm^{-3}$ and $[O_2]_0$ = 5 × $10^{18}$ molecule $cm^{-3}$ and the reactions and rate coefficients at 298 K shown in Table S1 to obtain time profiles of $[CH_3O_2]$ and [ClO] which were then multiplied by the absorption cross sections at 250 nm: $\sigma_{CH3O2}$ = 3.8 × $10^{-18}$ $cm^2$ $molecule^{-1}$ [17] and $\sigma_{ClO}$ = 3.5 × $10^{-18}$ $cm^2$ $molecule^{-1}$.[11]

## S2. Details of determination of the branching ratios in the $CH_3O_2$ self-reaction as a function of temperature

The branching ratio of the channel producing $CH_3O$, $r_{CH3O}$ was computed substituting the concentrations of $CH_2O$ and $CH_3OH$ measured using FTIR in the first few minutes of the experiments with the lamps turned on in equation S4 (equation 8 in the main text).

$$r_{CH3O} = \frac{[CH_2O]_{overall} - [CH_3OH]}{[CH_2O]_{overall} + [CH_3OH]} \qquad \text{(S4)}$$

Here $[CH_2O]_{overall} = [CH_2O]_a + [CH_2O]_b$, where $[CH_2O]_a = [CH_3OH]$ is $CH_2O$ produced by reaction (R4.a) and $[CH_2O]_b$ is formed by reaction (R4.b) followed by reaction (R2) (the reaction are notated as in the main text).

$$CH_3O_2 + CH_3O_2 \rightarrow CH_3OH + CH_2O + O_2 \qquad \text{(R4.a)}$$

$$CH_3O_2 + CH_3O_2 \rightarrow CH_3O + CH_3O + O_2 \qquad \text{(R4.b)}$$

$$CH_3O + O_2 \rightarrow CH_2O + HO_2 \qquad \text{(R2)}$$

Figure S5 shows examples of concentration–time profiles generated for the two reaction products, $CH_2O$ and $CH_3OH$. It can be noted that in the first 2–5 min of the reaction the increase in $[CH_2O]$ and $[CH_3OH]$ is linear.

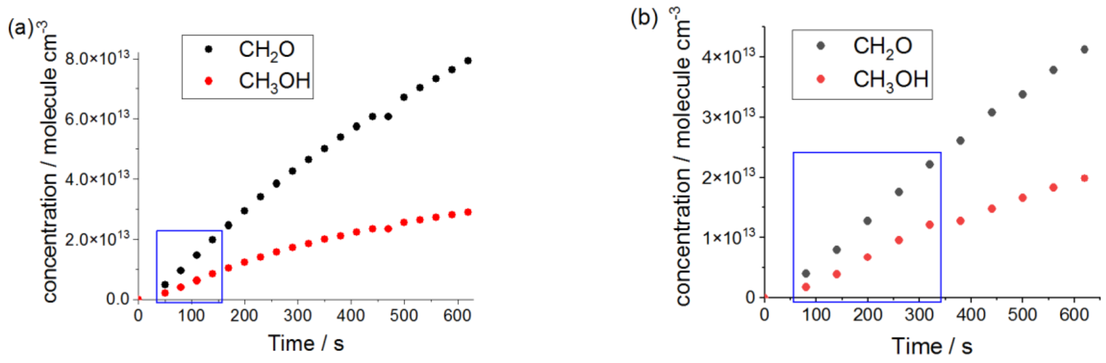


**Figure S5**. Examples of concentration–time profiles for $CH_2O$ (black) and $CH_3OH$ (red) obtained during the time with the lamps on at (a) 295 K and (b) 284 K using FTIR measurements. The blue frames shows the data used in equation S4 to determine the branching ratio of the channel producing $CH_3O$ of the $CH_3O_2$ self-reaction: (a) $r_{CH3O} = 0.40 \pm 0.02$ and (b) $r_{CH3O} = 0.31 \pm 0.03$ (statistical errors at $2\sigma$ level).

Numerical simulations performed using reaction R6 ($Cl_2$ photolysis) and the reactions shown in Table S1 at 298 K show that, after about 25 s induction time [$CH_2O$] and [$CH_3OH$] increase linearly in time at the beginning of the reaction (Figure S6).

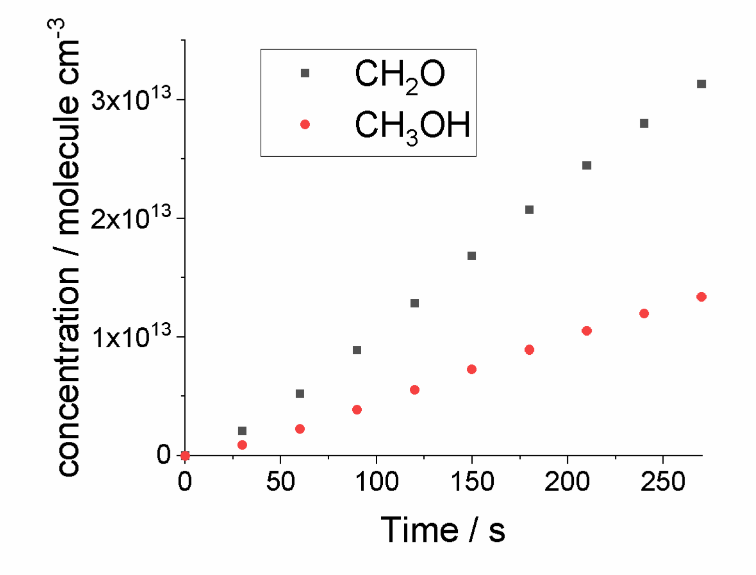


**Figure S6**. Examples of concentration–time profiles for $CH_2O$ (black) and $CH_3OH$ (red) at 298 K obtained by numerical simulations using $Cl_2$ photolysis (R6) followed by the reaction system described in Table S1.

Figure S7 shows the values generated for $r_{CH3O}$ at two temperatures, 268 K and 323 K, using $[CH_2O]_{overall}$ and [$CH_3OH$] measured in the first minutes of the reaction. It can be noted that there is no trend of $r_{CH3O}$ with time over this duration suggesting that the secondary reactions of $CH_2O$ and $CH_3OH$ are negligible. This is the case for all temperatures studied.

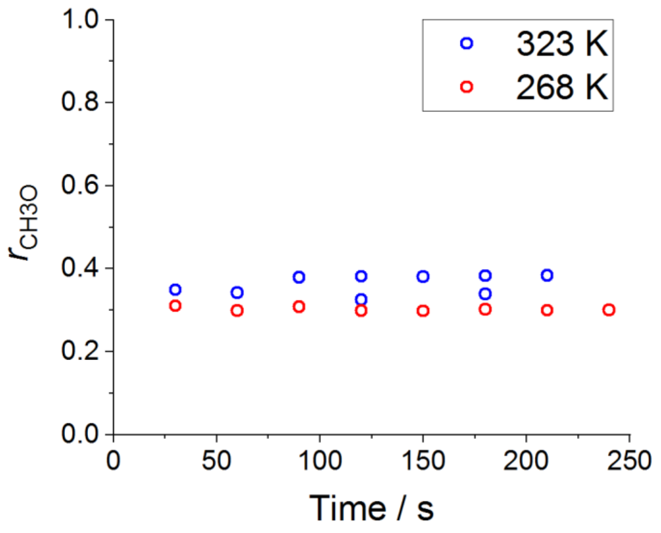


**Figure S7.** Branching ratio of the channel producing $CH_3O$ of the $CH_3O_2$ self-reaction (R4.b), $r_{CH3O}$ = 0.30 ± 0.01 at 268 K and $r_{CH3O}$ = 0.37 ± 0.04 at 323 K (statistical errors at $2\sigma$ level).

Table S2 shows the mean values calculated for $r_{CH3O}$ at the temperatures used.

**Table S2**. Mean values obtained for the branching ratio of the channel producing $CH_3O$ (R4.b), $r_{CH3O}$. The errors represent overall uncertainties calculated as the sum in quadrature of the statistical and systematic errors and are quoted at $2\sigma$ level.

| **Temperature / K** | $\boldsymbol{r_{CH3O}}$ |
|---|---|
| 268 | 0.30 ± 0.04 |
| 284 | 0.32 ± 0.05 |
| 295 | 0.34 ± 0.05 |
| 323 | 0.37 ± 0.05 |
| 344 | 0.43 ± 0.06 |

## References

1. Sander, S. P.; Watson, R. T., Kinetic studies of the reactions of $CH_3O_2$ with NO, $NO_2$ and $CH_3O_2$ at 298 K. *J. Phys. Chem.* **1980,** *84* (13), 1664-1674.

2. Sander, S. P.; Watson, R. T., Temperature dependence of the self-reaction of $CH_3O_2$ radicals. *J. Phys. Chem.* **1981,** *85* (20), 2960-2964.

3. McAdam, K.; Veyret, B.; Lesclaux, R., UV absorption spectra of $HO_2$ and $CH_3O_2$ radicals and the kinetics of their mutual reactions ant 298 K. *Chem. Phys. Lett.* **1987,** *133* (1), 39-44.

4. Kurylo, M. J.; Wallington, T. J., The temperature dependence of the rate constant for the gas phase disproportionation reaction of $CH_3O_2$ radicals. *Chem. Phys. Lett.* **1987,** *138* (6), 543-547.

5. Lightfoot, P. D.; Lesclaux, R.; Veyret, B., Flash photolysis study of the $CH_3O_2$ + $CH_3O_2$ reaction: Rate constants and branching ratios from 248 to 573 K. *J. Phys. Chem.* **1990,** *94* (2), 700-707.

6. Atkinson, R.; Baulch, D. L.; Cox, R. A.; Crowley, J. N.; Hampson, R. F.; Hynes, R. G.; Jenkin, M. E.; Rossi, M. J.; Troe, J., Evaluated kinetic and photochemical data for atmospheric chemistry: Volume II - gas phase reactions of organic species. *Atmos. Chem. Phys.* **2006,** *6*, 3625-4055.

7. Parker, J. K.; Payne, W. A.; Cody, R. J.; Nesbitt, F. L.; Stief, L. J.; Klippenstein, S. J.; Harding, L. B., Direct measurement and theoretical calculation of the rate coefficient for $Cl+CH_3$ in the range from T=202-298 K. *J. Phys. Chem. A* **2007,** *111* (6), 1015-1023.

8. Eskola, A. J.; Timonen, R. S.; Marshall, P.; Chesnokov, E. N.; Krasnoperov, L. N., Rate constants and hydrogen isotope substitution effects in the $CH_3$+HCl and $CH_3+Cl_2$ reactions. *J. Phys. Chem. A* **2008,** *112* (32), 7391-7401.

9. Maricq, M. M.; Szente, J. J.; Kaiser, E. W.; Shi, J. C., Reaction of chlorine atoms with methylperoxy and ethylperoxy radicals. *J. Phys. Chem.* **1994,** *98* (8), 2083-2089.

10. Atkinson, R.; Baulch, D. L.; Cox, R. A.; Crowley, J. N.; Hampson, R. F.; Hynes, R. G.; Jenkin, M. E.; Rossi, M. J.; Troe, J., Evaluated kinetic and photochemical data for atmospheric chemistry: Volume III - gas phase reactions of inorganic halogens. *Atmos. Chem. Phys.* **2007,** *7*, 981-1191.

11. Burkholder, J. B.; Sander, S. P.; Abbatt, J. P. D.; Barker, J. R.; Cappa, C.; Crounse, J. D.; Dibble, T. S.; Huie, R. E.; Kolb, C. E.; Kurylo, M. J.; Orkin, V. L.; Percival, C. J.; Wilmouth, D. M.; Wine, P. H., Chemical kinetics and photochemical data for use in atmospheric studies - Evaluation number 19. available at: http://jpldataeval.jpl.nasa.gov/, last access: 10 May 2022, 2020.

12. Pilling, M. J.; Smith, M. J. C., A laser flash-photolysis study of the reaction $CH_3+O_2$-> $CH_3O_2$ at 298 K. *J. Phys. Chem.* **1985,** *89* (22), 4713-4720.

13. Heicklen, J., The decomposition of alkyl nitrites and the reactions of alkoxyl radicals. *Adv. Photochem.* **1988,** *14*, 177-272.

14. Hassinen, E.; Koskikallio, J., Flash-photolysis of methyl acetate in gas phase - products and rate constants of reactions between methyl, methoxy and acetyl radicals. *Acta Chem. Scand., Ser. A* **1979,** *33* (8), 625-630.

15. Onel, L.; Brennan, A.; Seakins, P. W.; Whalley, L.; Heard, D. E., A new method for atmospheric detection of the $CH_3O_2$ radical. *Atmos. Meas. Tech.* **2017,** *10* (10), 3985-4000.

16. Winiberg, F. A. F.; Dillon, T. J.; Orr, S. C.; Gross, C. B. M.; Bejan, I.; Brumby, C. A.; Evans, M. J.; Smith, S. C.; Heard, D. E.; Seakins, P. W., Direct measurements of OH and other product yields from the $HO_2$ + $CH_3C(O)O_2$ reaction. *Atmos. Chem. Phys.* **2016,** *16* (6), 4023-4042.

17. Tyndall, G. S.; Cox, R. A.; Granier, C.; Lesclaux, R.; Moortgat, G. K.; Pilling, M. J.; Ravishankara, A. R.; Wallington, T. J., Atmospheric chemistry of small organic peroxy radicals. *J. Geophys. Res. -Atmos.* **2001,** *106* (D11), 12157-12182.
